# Geography vs. past climate: the drivers of population genetic structure of the Himalayan langur

**DOI:** 10.1186/s12862-022-02054-1

**Published:** 2022-08-15

**Authors:** Kunal Arekar, Neha Tiwari, Sathyakumar Sambandam, Mehreen Khaleel, Praveen Karanth

**Affiliations:** 1 Centre for Ecological Sciences, Indian Institute of Science, Bengaluru, India; 2 Wildlife Institute of India, Dehradun, India; 3 Wildlife Research and Conservation Foundation, Srinagar, Jammu and Kashmir India

**Keywords:** Genetic diversity, Phylogeography, Riverine barrier effect, Past climate, Heterogenous landscape

## Abstract

**Background:**

Contemporary species distribution, genetic diversity and evolutionary history in many taxa are shaped by both historical and current climate as well as topography. The Himalayas show a huge variation in topography and climatic conditions across its entire range, and have experienced major climatic fluctuations in the past. However, very little is known regarding how this heterogenous landscape has moulded the distribution of Himalayan fauna. A recent study examined the effect of these historical events on the genetic diversity of the Himalayan langurs in Nepal Himalaya. However, this study did not include the samples from the Indian Himalayan region (IHR). Therefore, here we revisit the questions addressed in the previous study with a near complete sampling from the IHR, along with the samples from the Nepal Himalaya. We used the mitochondrial Cytochrome-*b* (Cyt-*b*, 746 bp) region combined with multiple phylogeographic analyses and palaeodistribution modelling.

**Results:**

Our dataset contained 144 sequences from the IHR as well as the Nepal Himalaya. Phylogenetic analysis showed a low divergent western clade nested within high divergent group of eastern lineages and in the network analysis we identified 22 haplotypes over the entire distribution range of the Himalayan langurs. Samples from the Nepal Himalaya showed geographically structured haplotypes corresponding to different river barriers, whereas samples from IHR showed star-like topology with no structure. Our statistical phylogeography analysis using diyABC supported the model of east to west colonisation of these langurs with founder event during colonisation. Analysis of demographic history showed that the effective population size of the Himalayan langurs decreased at the onset of last glacial maximum (LGM) and started increasing post LGM. The palaeodistribution modelling showed that the extent of suitable habitat shifted from low elevation central Nepal, and adjoining parts of north India, during LGM to the western Himalaya at present.

**Conclusion:**

The current genetic diversity and distribution of Himalayan langurs in the Nepal Himalaya has been shaped by river barriers, whereas the rivers in the IHR had relatively less time to act as a strong genetic barrier after the recent colonisation event. Further, the post LGM expansion could have had confounding effect on Himalayan langur population structure in both Nepal Himalaya and IHR.

**Supplementary Information:**

The online version contains supplementary material available at 10.1186/s12862-022-02054-1.

## Background

Accumulation of genetic variation across different populations of a species could be attributed to changes in geography and climate via a combined effect of genetic drift, gene flow and selection [1–3]. Geographical barriers such as mountain ranges and rivers; environmental barriers; and certain behavioural aspects of the taxa such as philopatry, dispersal ability, etc. can prevent gene flow, and over time create population genetic structures [4, 5].

Rivers are considered as long-term barriers to gene flow and therefore plays an important role in speciation and divergence [6–8]. Wallace [9] noted that many south American primate species are separated by large rivers in the Amazon basin, and that different species of primates are restricted to opposite banks of the river and never cross it. The ability of a river to act as a barrier depends on various physical attributes of the river such as the size, the amount of discharge and speed of the water flow; further the dispersal ability, ecology and body size of the organism also plays an important role [10, 11]. The effects of rivers as barriers have been studied in many taxa of plants, amphibians, reptiles, mammals and birds [12–21]. In primates, these studies of riverine barrier effect have mostly been confined to Amazonian and African taxa [4, 10, 11, 22–24], with only a handful of studies in Asian primates [25–29]. Apart from the geographical barriers, Climatic fluctuations are also known to have an impact on the population genetic structure and demography [30–33]. Quaternary glaciation periods, such as the last glacial maximum (LGM), substantially influenced how genetic variation is spatially distributed in many species across the globe [31, 34]. Repeated periods of glaciation, especially in higher latitudes, resulted in range expansion—in the case of species adapted to cold climate; and range contraction—in the species adapted to warm climate [31, 34–45]. In the latter case, species may undergo range expansion and exponential population growth after the end of the glacial period [46, 47].

The Himalayan range is a globally recognised biodiversity hotspot with high levels of endemism and a complex geography, topography and climate [48]. It extends 2500 kms in length from west to east and its width varies between 350 km in the west and 150 km in the east [49]. Given these, there are very few studies testing the effect of such a complex topography and climatic fluctuations on the distribution and demography of species found in the Himalayas [28, 29, 50, 51]. Himalayan langur, (Primate: Colobinae) is a widely distributed alloprimate found in most parts of the Himalayas. It is distributed in Nepal and parts of India, Pakistan and Bhutan [52], the Black mountain range and the Sunkosh river in Bhutan forms the easternmost distribution limit for this species [53]. Until recently, there was much ambiguity in the taxonomy of these langurs owing to presence of multiple classification schemes, however IUCN recognised three species—*Semnopithecus ajax*, *Semnopithecus schistaceus* and *Semnopithecus hector*, as per the classification scheme proposed by Groves [54]. Nevertheless, there was no consensus within the scientific community on the use of any one classification scheme and the names proposed by all the schemes were considered to be valid and were used by different studies [28, 55–60]. This use of different taxonomic names for the same species can create serious issues while interpreting results from these studies, therefore, it was important to resolve the taxonomy of this group. In this context, a recent systematic study resolved the taxonomy of these langurs using an integrative approach based on three lines of evidence from molecular, morphological and ecological data. The authors have now subsumed the three recognised species into a single widespread species *Semnopithecus schistaceus* Hodgson, 1840 [52].

Another study investigated the role of Himalayan rivers as well as past climate in shaping the distribution of Himalayan langur in Nepal Himalaya [28]. They found that Himalayan langur populations (they used the name *S*. *entellus* in their paper) in the Nepal Himalayas were isolated into six major clades by the snow-fed Himalayan rivers suggesting the role of these rivers as a barrier to gene flow. Further, their demographic analysis showed that the Himalayan langur populations in Nepal started declining with the onset of the last glacial maximum (LGM) i.e., ~ 22,000 years before present (YBP). For their study, they used two mitochondrial genes—Cytochrome *b* (Cyt *b*) and hyper variable region (HVR) I. However, this study did not include any samples from the Indian Himalayan region (IHR) which constitutes a major part of the Himalayan langur distribution zone.

In this study, we revisit the questions addressed by Khanal et al. [28] with near complete sampling from India. Along with the samples from the Nepal Himalaya [28], we have expanded the sampling into the Indian Himalayan region and included three river valleys from this region—Sutlej river valley, Bhagirathi river valley, and Mahakali/Sharda (hereafter Mahakali) river valley. Given this extensive sampling, covering the entire distribution range of the Himalayan langur in this study, we first investigate the role of major Himalayan rivers in shaping the population structure of Himalayan langurs. For this, we have included all the river valleys studied by Khanal et al. [28] (except the river Trishuli since it did not act as barrier for gene flow as per [28]) along with three river valleys from the IHR west of Nepal. Additionally, given the wider sampling of Himalayan langurs in this study we revisit the role of past climate, specifically the last glacial maximum (LGM), in shaping the contemporary distribution of the Himalayan langur. Finally, we investigate the purported westward expansion of the Himalayan langur from Nepal and Sikkim. In the molecular phylogenetic tree of Arekar et al. [52], a low divergent western clade was nested within high divergent eastern lineages suggesting a potentially east to west dispersal. Therefore, we considered the western clade as one population namely the western metapopulation (WMP) and the high divergent eastern lineages as another population i.e., the eastern metapopulation (EMP). To test the east to west dispersal hypothesis, we used a model-based hypothesis testing in a Bayesian framework using Approximate Bayesian computation (ABC) analysis implemented in DIY ABC v2.1.0 [61].

## Results

### Phylogenetic analysis

**Fig. 1:**
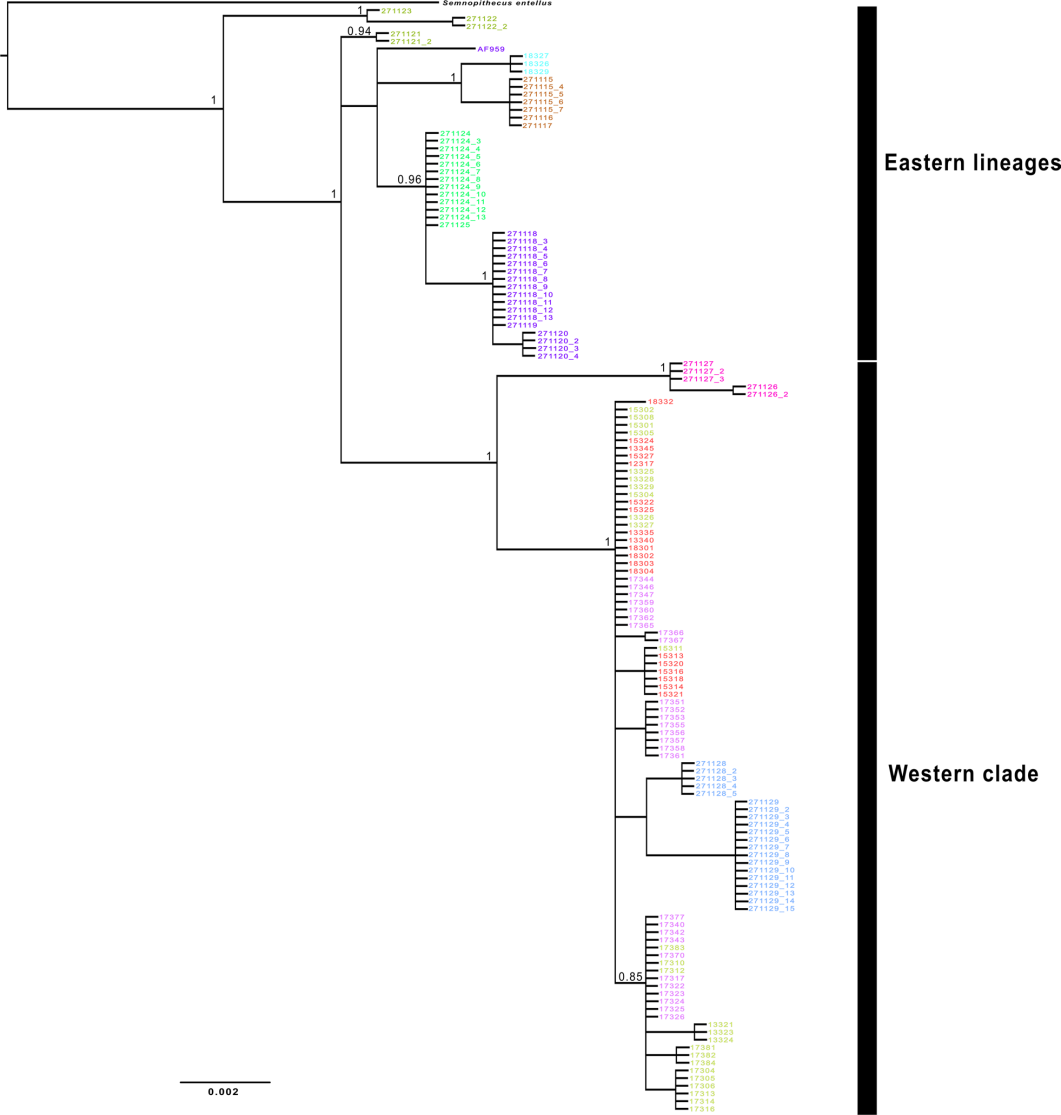
Bayesian phylogeny of Himalayan langur for mitochondrial cytochrome *b* (Cyt-*b*) gene. Numbers at the node indicate Bayesian posterior probability (BPP) values. Node support values > 0.75 are shown. Node support values are shown only for major clades. The colours for the tip taxa correspond to the colours of the 10 populations in the haplotype network in Fig. 2

The final dataset contained 746 bp of 145 Himalayan langur sequences, out of these 51 were sequenced in this study; 25 sequences were downloaded from Arekar et al. [52]; 67 sequences from Khanal et al. [28]; 1 sequence from Karanth et al. [62] and one *S*. *entellus* sequence was used as an outgroup (Additional file 4: Table S1) [28, 52, 62]. HKY + G substitution model was selected for this analysis. In both the Bayesian (Fig. 1) and ML tree (Additional file 1: Fig. S1), we found a well-supported clade—the western clade, consisting of haplotypes from the western Himalayas (west of the river KaliGandaki), which was nested within samples from eastern Himalayas (east of the river KaliGandaki) i.e., the eastern lineages. Both the trees showed similar topology wherein all the major clades were retrieved. The only exception was in the Bayesian tree (Fig. 1), the MH271128 and MH271129 sequences were placed within the clade containing samples from the Indian Himalayan region (IHR), whereas in the ML tree (Additional file 1), these two lineages were sister to the IHR clade. The position of the MH271121 clade was also different in the Bayesian and ML trees. Our divergence dating analysis showed that the western clade (i.e. WMP) diverged from the eastern lineages (i.e. EMP) at around 0.52 mya (CI 0.28–0.78). Further, the population in IHR diverged from the rest of the Himalayan langurs at 0.29 mya (CI 0.16–0.44) (Additional file 2).

### Phylogeographic analyses

For these analyses, we removed the outgroup sequence from our dataset and the sequence length was shortened to 645 bp due to removal of sites with missing data. So, the final dataset for these analyses contained 144 sequences.

#### Network analysis, genetic diversity, and population genetic structure

**Fig. 2:**
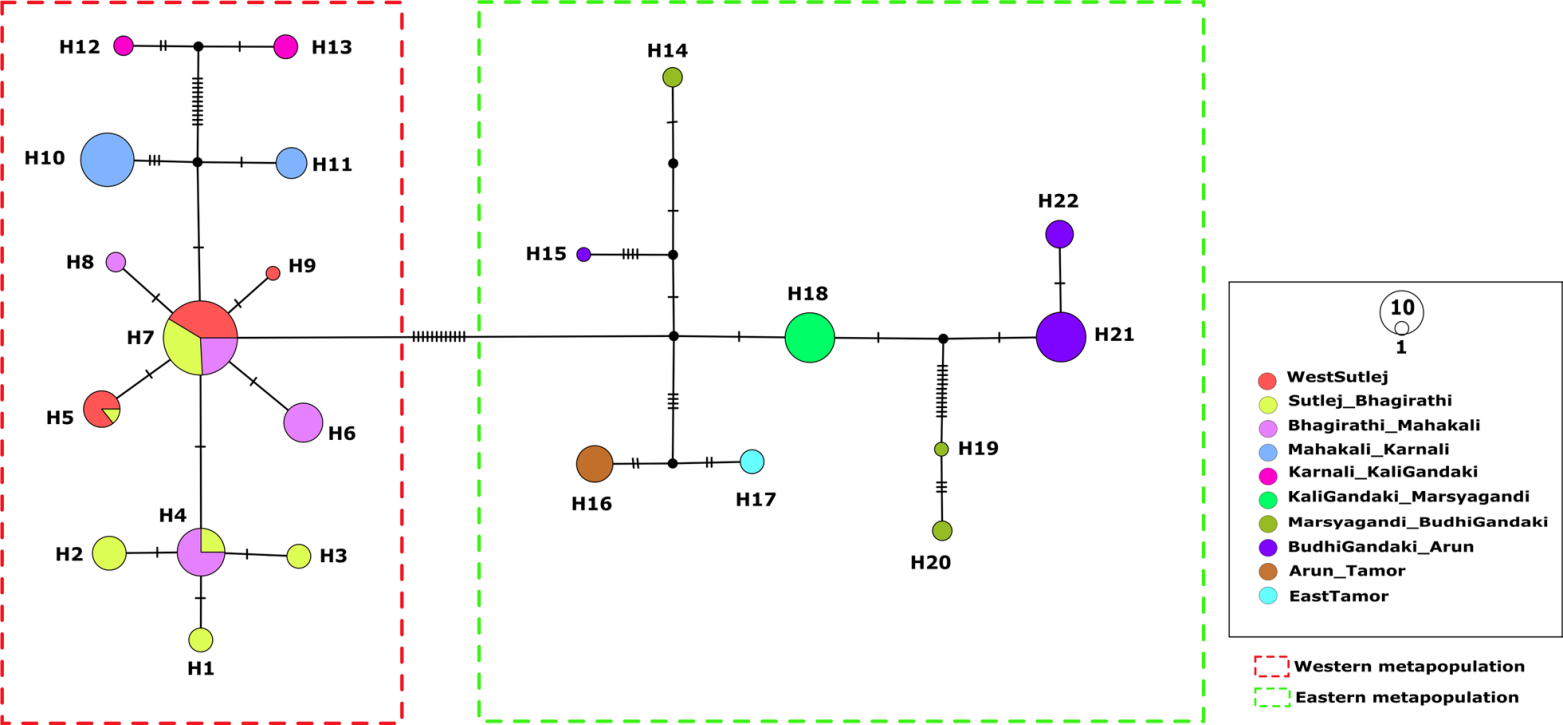
Haplotype network for the Himalayan langurs using Cyt-*b* sequence. The coloured circles represent extant haplotypes while the black circles are inferred intermediate haplotypes not sampled in this study. The sizes of the haplotypes are proportional to their frequency i.e., the number of individuals that constitute those haplotypes. The bars on the link between the circles stand for the number of substitutions between those haplotypes. The dotted red and green lines indicate the two larger groups—WMP and EMP, respectively

**Fig. 3:**
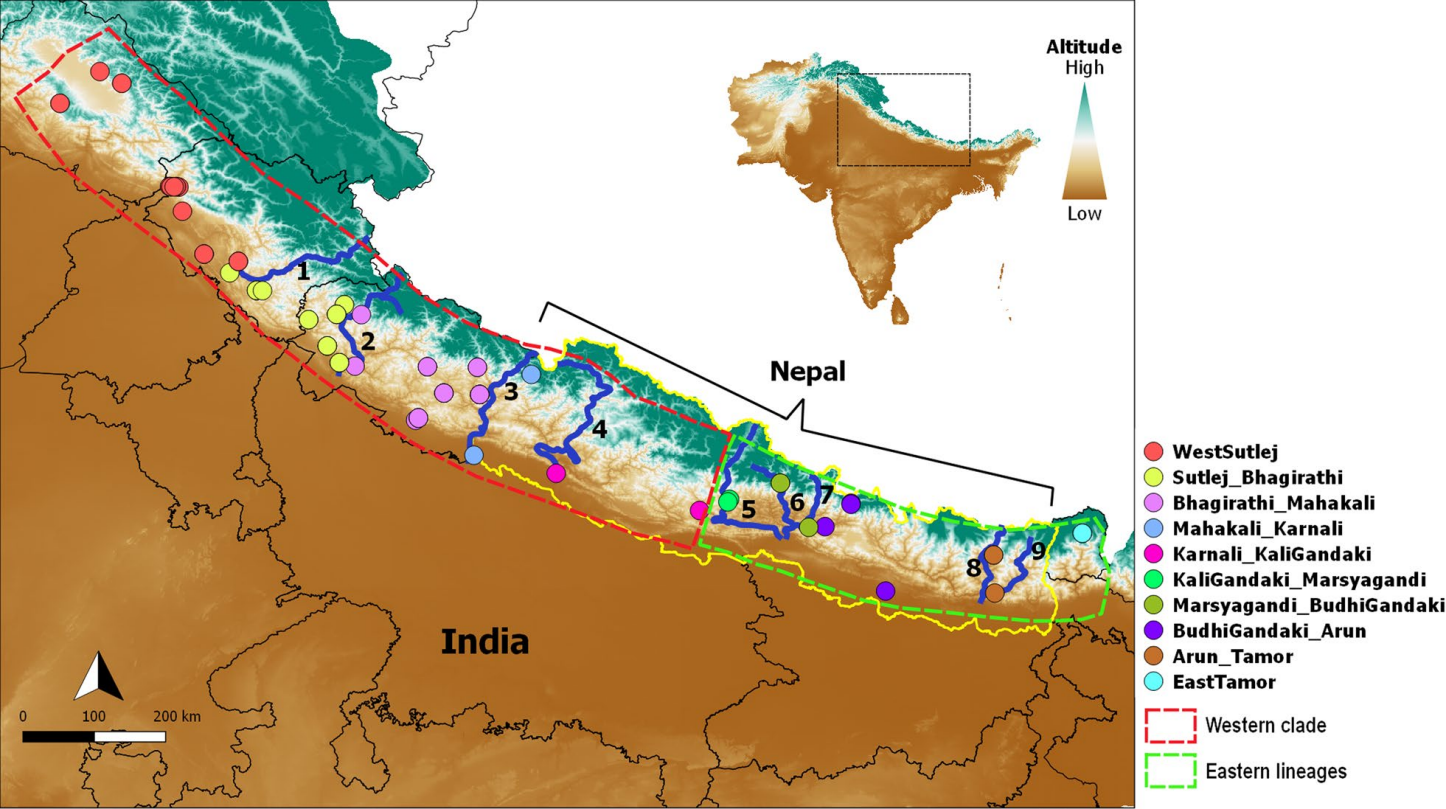
Diagram showing the 10 populations of Himalayan langurs separated by the nine river valleys; 1—River Sutlej, 2—River Bhagirathi, 3—River Mahakali, 4—River Karnali, 5—River KaliGandaki, 6—River Marsyagandi, 7—River BudhiGandaki, 8—River Arun, 9—River Tamor. The colours of these populations correspond with the haplotype colours in Fig. 2﻿. The yellow borderline demarcates the country of Nepal, its extent is also shown in the map using a square bracket

The network analysis of 144 sequences yielded 22 haplotypes across the distribution zone of the Himalayan langurs (Fig. 2). Two clusters can be observed in the network, one consisting of the WMP (H1–H13) i.e., all the sequences from the western Himalayan region (west of the river KaliGandaki), and the other consisting of the EMP (H14–H22) i.e., all the sequences from the eastern Himalayan region (east of the river KaliGandaki). These two clusters are spatially delineated by the river Kali Gandaki. Within the western population, one high frequency haplotype (H7, n = 29) was observed which consisted majority of the individuals from the western region. The haplotypes H10 and H11 were separated from the rest of the samples of the IHR by river Mahakali; (these two haplotypes correspond to the WB clade as labelled in Khanal et al. [28]). The haplotypes H12 and H13 were also seen forming a separate cluster separated from the rest by river Karnali, these two haplotypes correspond to the clade WA in Khanal et al. Other than these, the samples from the IHR showed very little structure across the river valleys. On the contrary, the eastern population showed highly structured haplotypes separated by the river valleys in the Nepal Himalayas as shown in Fig. 3. The river Marsyagandi separated the haplotype H18 (which corresponds to clade CC in Khanal et al. [28]) from the haplotypes H19 and H20 (these haplotypes correspond to clade CB in Khanal et al. [28]). And the river Budhi Gandaki separated the haplotypes H19 and H20 from the haplotypes H21 and H22 (these haplotypes correspond to clade CA in Khanal et al. [28]). Further, the rivers Arun and Tamor separated the haplotypes H16 and H17, respectively. Here the haplotype H16 corresponds to the clade EA in Khanal et al. [28]. In this study, the haplotype H14 is placed in the population Marsyagandi_BudhiGandaki (My–Bg) based on the geographical proximity however, in the haplotype network it can be seen as an outlier; and it corresponds to the haplotype H19 in Khanal et al. [28]. This pattern seen in the haplotype network analysis is similar to the results from our phylogenetic analysis (Fig. 1).

The nucleotide diversity (π) of the My–Bg population was highest (0.015), in fact it was equivalent to all the populations combined together; the haplotype diversity (H) was also high (0.8) (Table 1). The populations to the west and to the east of the My–Bg has low π and H values. The genetic diversity data for three populations; KaliGandaki_Marsyagandi (Kg–My), Arun_Tamor (A–T) and EastTamor (E-T) was not obtained due to the absence of polymorphic sites in the nucleotide sequences.

**Table 1:** DNA polymorphism and genetic diversity of different populations of the Himalayan langurs

Population	S	π	H	Hd	Tajima’s D	Fu’s F	R_2_
WestSutlej (n = 19)	2	0.0008	3	0.526	− 0.045	− 0.032	0.1596
Sutlej_Bhagirathi (n = 26)	5	0.002	6	0.788	0.147	− 0.903	0.1357
Bhagirathi_Mahakali (n = 28)	3	0.002	4	0.722	0.886	0.471	0.1759
Mahakali_Karnali (n = 20)	4	0.002	2	0.395	1.159	4.399	0.1974
Karnali_KaliGandaki (n = 5)	3	0.003	2	0.6	1.572	2.429	0.3000
KaliGandaki_Marsyagandi (n = 13)	0	–	–	–	–	–	–
Marsyagandi_BudhiGandaki (n = 5)	16	0.015	3	0.8	1.832	3.709	0.3000
BudhiGandaki_Arun (n = 18)	9	0.002	3	0.451	**− 1.844**	1.664	0.2046
Arun_Tamor (n = 7)	0	–	–	–	–	–	–
EastTamor (n = 3)	0	–	–	–	–	–	–
All individuals (n = 144)	60	0.015	22	0.915	− 0.396	2.340	0.0796
WMP (n = 98)	27	0.005	13	0.855	− 1.021	− 0.224	0.0638
EMP (n = 46)	30	0.009	9	0.818	− 0.591	3.229	0.0961

Values in bold shows significance at P < 0.05

*S* polymorphic sites, *π* nucleotide diversity, *H* no. of haplotypes, *Hd* haplotype diversity, *n* no. of individuals, *R*_*2*_ Ramos-Onsin’s and Rozas’s R_2_ statistic

The pairwise F_ST_ values between populations across WMP and EMP were higher than between populations within WMP or EMP (Table 2). Further, the F_ST_ values were low among populations that were geographically closer whereas they were higher in spatially distant populations.

**Table 2:** Comparisons of pairs of population samples—population pairwise Fst (below diagonal), average number of pairwise difference between populations (above diagonal) and average number of pairwise differences within populations (diagonal elements in bold) among the different populations of Himalayan langurs calculated by distance method

	WMP					EMP				
	W–S	S–B	B–M	M–Kr	Kr–Kg	Kg–My	My–Bg	Bg–A	A–T	E-T
WMP										
W–S	**0.561**	1.421	1.118	3.868	13.768	13.368	19.968	15.702	18.368	18.368
S–B	0.300	**1.378**	1.374	4.577	14.477	14.077	20.677	16.410	19.077	19.077
B–M	0.263	0.115	**1.056**	4.250	14.150	13.750	20.350	16.083	18.750	18.750
M–Kr	0.721	0.679	0.698	**1.579**	14.900	15.000	20.700	16.000	21.500	21.500
Kr–Kg	0.942	0.900	0.917	0.891	**1.800**	19.200	24.600	21.533	23.200	24.200
EMP										
Kg–My	0.975	0.935	0.948	0.936	0.976	**0.000**	10.600	2.444	7.000	7.000
My–Bg	0.876	0.863	0.879	0.841	0.768	0.728	**9.600**	10.889	16.200	16.800
Bg–A	0.943	0.919	0.929	0.911	0.937	0.710	0.682	**1.255**	9.333	9.333
A–T	0.977	0.943	0.955	0.945	0.969	1.000	0.753	0.903	**0.000**	4.000
E-T	0.973	0.934	0.948	0.935	0.951	1.000	0.637	0.884	1.000	**0.000**

W-S: WestSutlej; S–B: Sutlej_Bhagirathi; B–M: Bhagirathi_Mahakali; M–Kr: Mahakali_Karnal; Kr–Kg: Karnali_KaliGandaki; Kg–My: KaliGandaki_Marsyagandi; My–Bg: Marsyagandi_BudhiGandak; Bg–A: Budhigandaki_Arun; A–T: Arun_Tamor; E-T: EastTamor

#### Demographic history

**Fig. 4:**
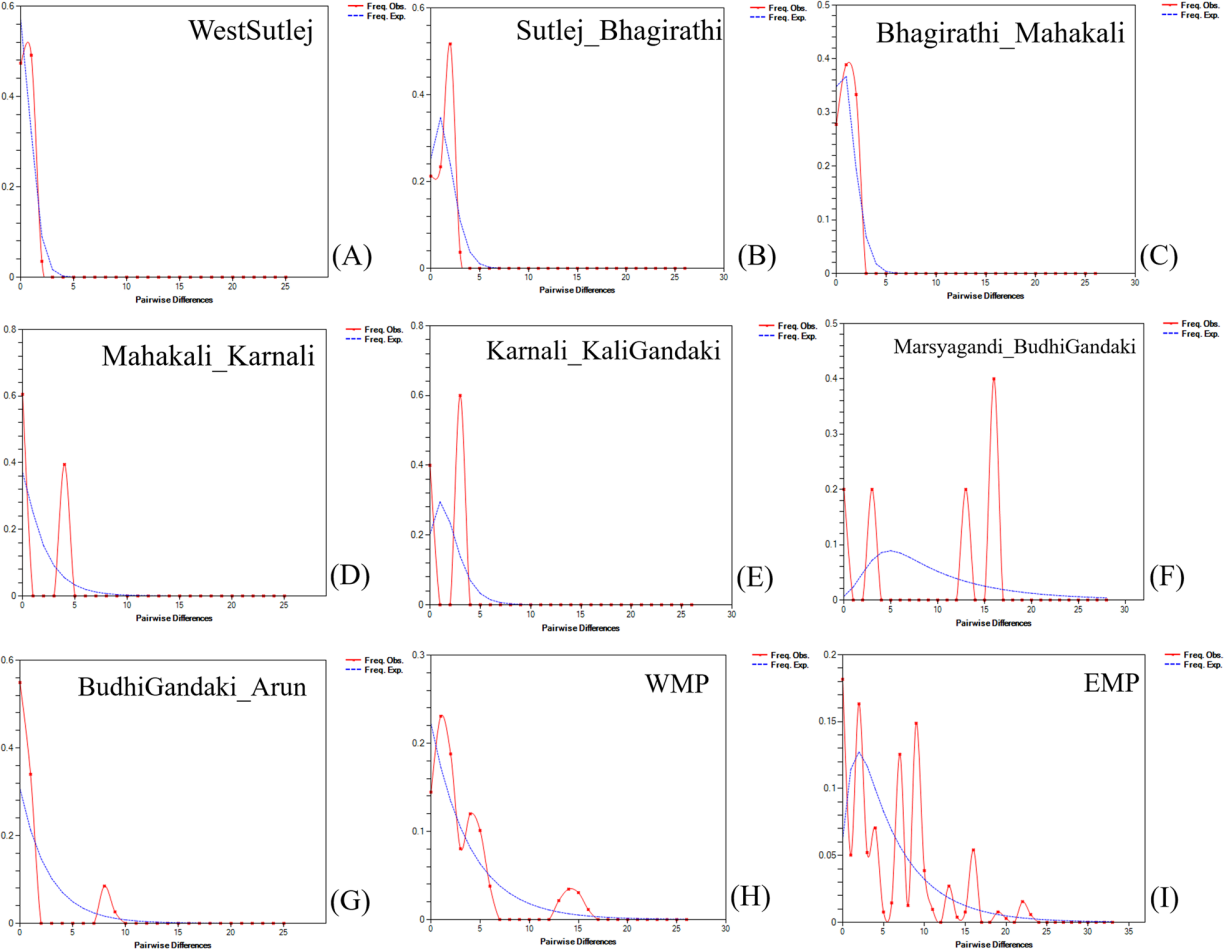
Results of mismatch distribution carried out on Cyt-*b* data obtained from the Himalayan langurs. The graphs show pairwise differences between sequences (X-axes) plotted against the frequency of those differences to generate the distributions. The blue dotted line indicates expected distribution under a population growth-decline model. *WMP *western metapopulation, *EMP *eastern metapopulation. The panels **A**–**I** represent individual mismatch graphs for different populations, the population names are mentioned on the top right corner of each graph

Our mismatch distribution analyses for populations in the WMP and the EMP, each produced different patterns (Fig. 4). The EMP yielded a multimodal distribution of the observed data (Fig. 4I). Within the EMP, the population My–Bg showed a multimodal distribution (Fig. 4F); all the other population either showed a unimodal distribution, or a bimodal distribution closer to y-axis.

**Fig. 5:**
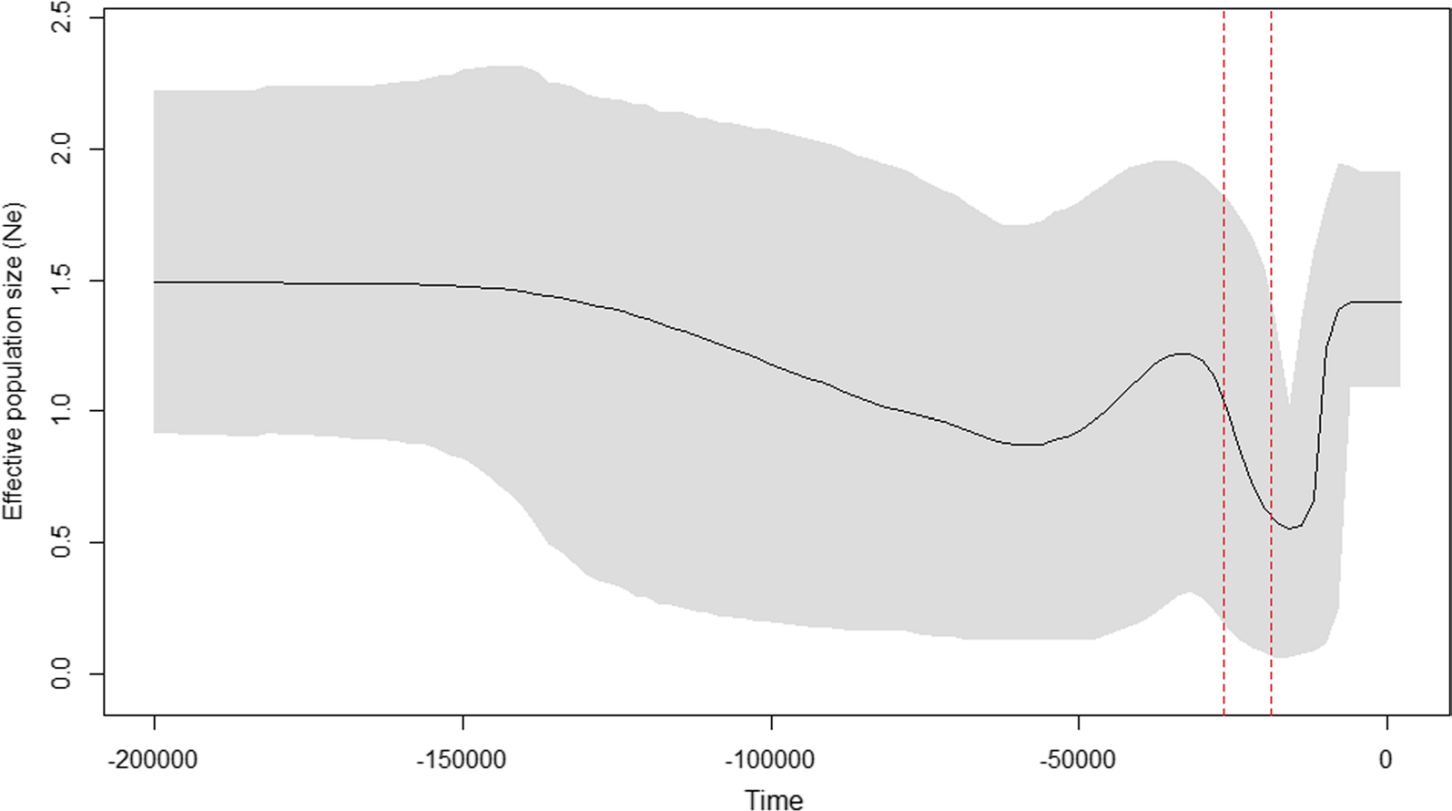
Bayesian skyline plot reconstructed using Cyt-*b* gene for the Himalayan langur (*S*. *schistaceus*). This plot shows the changes in effective population size (Ne) through time. X-axis is time in years before present and Y-axis is the estimated effective population size. The solid line indicates the median Ne; the grey shaded area shows the 95% highest posterior density (HPD) intervals; vertical red dotted lines denotes the range of LGM from 26.5 to 19 ka as per [63]

The test of neutrality to check for excess of rare mutations as evidence for population expansion was not significant, except for the population BudhiGandaki_Arun (Bg–A) which showed a significantly negative Tajima’s D value (− 1.844, p < 0.05) (Table 1). The Bayesian skyline plot analysis for the overall dataset showed that the effective population size (Ne) of the Himalayan langurs decreased during the LGM, and it started increasing post LGM ultimately reaching its current population size (Fig. 5). The separate Bayesian skyline plots for WMP and EMP were not informative.

#### Statistical phylogeography

**Fig. 6:**
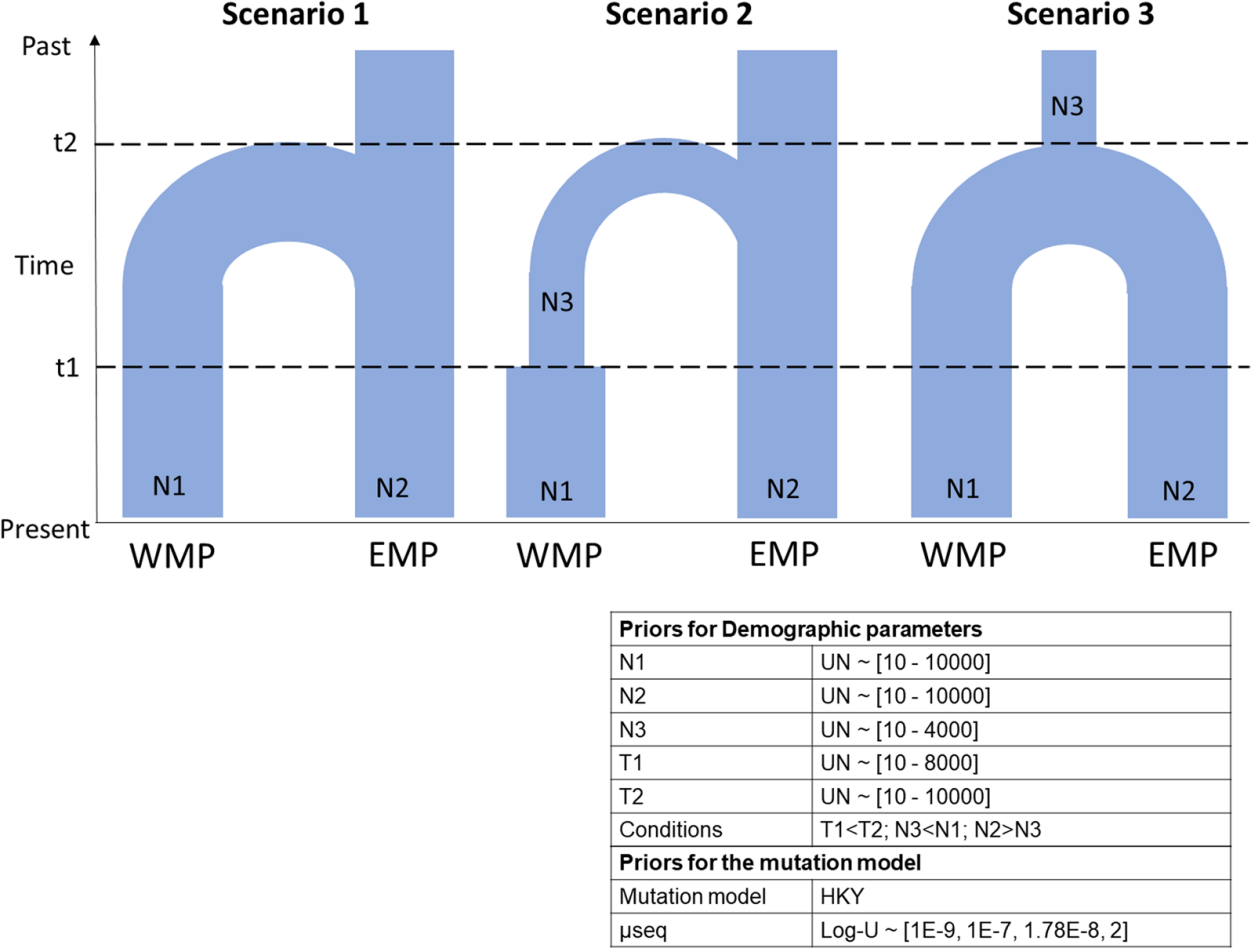
Alternative scenarios of demographic history of the Himalayan langur (*S. schistaceus*) tested using ABC analysis in DIYABC. Detailed explanation is mentioned in “Demographic history” in “Methods” section. Time has been measured in generations before present. The table alongside this figure contains details of the model specified, prior distributions for the demographic parameters and the mutation model parameters. *WMP* western metapopulation, *EMP* eastern metapopulation

For the ABC analysis we tested three scenarios (Fig. 6), Scenario 1 hypothesised that WMP originates from EMP with no change in the effective population size; Scenario 2 hypothesised a founder event in WMP; and Scenario 3 assumed bottleneck in the ancestral population of WMP and EMP. Further details are explained in “Methods” section. Here, the second scenario was selected as the scenario of choice with highest posterior probabilities in all the summary statistics used. In this scenario, we hypothesised a founder event where few individuals from the EMP colonised the western Himalayas and the population size eventually increased to the current state (Fig. 6, scenario 2). Model checking too supported scenario 2, since among the three scenarios the simulated dataset under scenario 2 were closest to the observed values.

### Niche modelling using past climate layers

**Fig. 7:**
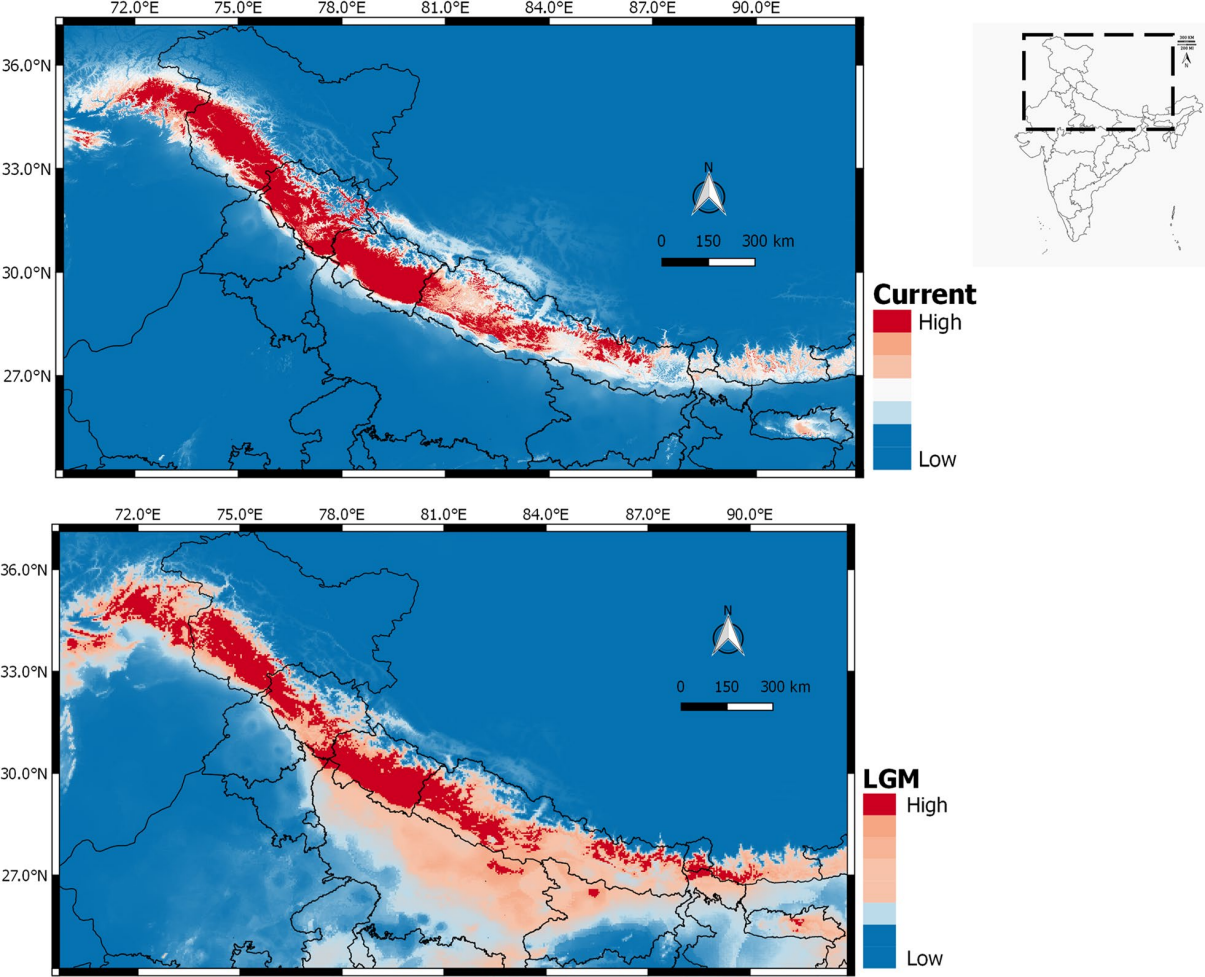
Ecological niche modelling projections of the Himalayan langur (*Semnopithecus schistaceus*). Top panel shows current distribution and the bottom panel shows potential distribution during LGM

The AUC values for the training and test data for the Himalayan langur dataset were 0.9772 and 0.9702, respectively, indicating that the potential distribution of the Himalayan langurs fits well with our data. Precipitation of the driest quarter (Bio 17) had the highest contribution to the model (45.1%) followed by annual mean temperature (Bio1; 26.2%) and precipitation seasonality (Bio15; 8.4%). The response curves reveal that Bio17 value of above 200, Bio 1 value between 80 and 120 and Bio 15 value in the range of 115–121 seem to be an ideal habitat for Himalayan langurs (Additional file 3). According to the palaeodistribution model the distribution of Himalayan langurs in the LGM was more towards south especially in the lowland Terai region of central Nepal and adjoining parts of northern India; however, the probability of distribution was moderate. Further, the probability of distribution of these langurs in the western Himalaya was higher for current time than during LGM (Fig. 7).

## Discussion

After decades of taxonomic confusion, the Himalayan langurs were recently assigned to a single species *Semnopethicus schistaceus* [52]. These langurs have a wide distribution in the Himalayas and therefore are a perfect model system to study the effect of landscape heterogeneity on genetic diversity and population genetic structure. The Himalayan langurs are threatened by habitat destruction—a result of human mediated climate change and human population expansion [64]. A recent study, using species distribution modelling, predicted range contraction of ~ 64% by 2050 under the GCMs RCP 4.5 and 8.5 [65]. Here, we investigated the drivers of population demography and genetic structure of the Himalayan langurs. Results from this study can inform the future management and conservation actions for this species.

### Genetic diversity and population structure

The study found that all the Himalayan langur samples, when pooled together, have a high haplotype diversity (Hd = 0.915) when compared to another temperate colobine *Rhinopithecus brelichi* (Hd = 0.457), and a low nucleotide diversity (π = 0.015) which is comparable to *R. brelichi* (π = 0.014) [66]. This combination of high haplotype diversity and low nucleotide diversity could be the result of population expansion after a recent bottleneck event or population expansion after a recent colonisation event [30]. To test for the riverine barrier hypothesis, we divided the Himalayan langur individuals into different populations demarcated by river valleys, here we found that the population My–Bg showed a high haplotype diversity (Hd = 0.8) and its nucleotide diversity was highest among all other populations (π = 0.015). This suggests that the My–Bg population which is demarcated by the river Marsyagandi and the river Budhi Gandaki in central Nepal, could either be a long-term stable population or it could also suggest that this population contains distinct haplotypes, probably originating from different sources. Rest of the populations showed a low π and Hd values, indicative of population expansion either after a recent bottleneck event or a recent colonisation event. Results from our pairwise F_ST_ analysis indicates that the WMP and EMP have been separated from each other longer than populations within WMP and EMP.

### Do himalayan rivers act as barriers?

Our phylogenetic tree with wider sampling is very similar to the tree in Arekar et al. [52] wherein a western clade with low divergence (corresponding to WMP) was nested within a larger phylogeny consisting of highly divergent sequences from eastern region (corresponding to EMP). The network analysis (Fig. 2), generated two major clusters separated by the river Kali Gandaki—the western cluster and the eastern cluster which corresponds to WMP and EMP, respectively (as seen in Fig. 1). The western cluster showed a star-like phylogeny which suggests shallow genetic structure and recent demographic expansion [67]. Whereas, the eastern cluster showed higher divergence among different haplotypes. The rivers in the IHR did not cause any structuring among different populations, in that haplotypes are shared across rivers. However, the rivers in the Nepal Himalaya i.e., Mahakali, Karnali, Kali Gandaki, Marsyagandi, Budhi Gandaki, Arun and Tamor, shaped the high genetic structure among different populations. Two populations within the WMP falls in the Nepal Himalaya i.e. Mahakali_Karnali (M–Kr) and Karnali–KaliGandaki (Kr–Kg), these two populations shows structured haplotypes across the river Karnali. Given that WMP are younger than the populations in the east i.e., EMP, it is plausible that the rivers in the IHR did not have enough time to act as barriers. Rivers have been shown to act as barriers to gene flow across different taxa [22, 31, 68], however the ability of river to act as barrier depends on various factors such as its size, speed of the flow of water, body size of the organism, and the dispersal ability of the organism [22]. The Kali Gandaki river is characterised by its strong water currents and it forms the deepest gorge in the world, therefore not surprising it forms a barrier against geneflow between the two populations—WMP and EMP, of Himalayan langurs.

### Effects of past glaciation events on demographic history

The effects of past climatic events, such as Pleistocene glaciation, on the distribution and demography of a species includes range contraction, range fragmentation, local extinction and resultant bottleneck [69, 70]. After the glaciation ends, species may undergo range expansion and exponential population growth [46, 47]. On the contrary, the habitats of taxa adapted to cold and dry conditions expands/persists during glaciation events [35–37, 44, 45].

In our analysis, the Tajima’s D, Fu’s F, and Ramos-Onsins and Rozas’s R_2_ statistic were not informative since the values for these indices were not significant in any of the population, except for the population Bg–A. However, it should be noted that these statistics by itself may not be very reliable for investigating the demographic history of a population and may depend on additional factors. So, for reliable predictions of demographic history, a combination of different indicators should be used [71]. In our mismatch distribution analysis, the EMP shows multimodal graph (Fig. 4I) which could either suggest that it is a long-term stable population or this population consists of haplotypes originating from more than one source. It should be noted that the mismatch distribution pattern is significantly affected by population structure [72, 73] and since haplotypes within EMP are highly structured (Fig. 2), we think that the mismatch analysis result here indicates the later. WMP also shows a multimodal graph but most likely it might be a result of population structure within WMP (Fig. 4H). We also performed mismatch analysis for individual populations (Fig. 4A–F); the three populations in the WMP which are distributed in the IHR showed a unimodal distribution which indicates population expansion (Fig. 4A–C). The populations M–Kr and Kr–Kg show a bimodal pattern which generally is an indicator of population under bottleneck, the data here is insufficient to support this result. And for the individual populations within the EMP, the populations My–Bg and Bg–A (Fig. 4F, G) showed a multimodal graph indicating that either these are long-term stable populations or the haplotypes they contain have originated from multiple source populations. Other populations within EMP did not show signatures of long-term stable population.

The Bayesian skyline plot indicated that the Himalayan langur population size was constant for a long period of time and started decreasing about ~ 25,000 years before present (YBP) i.e., after the onset of the last glacial maximum (LGM). After the LGM ended, the effective population size started increasing and reached the present-day estimate (Fig. 5). Our ABC analysis also supported the demographic expansion scenario in the WMP after it diverged from the EMP i.e., Scenario 2 (Fig. 6), which was supported by higher posterior probability. This suggests a founder effect during colonisation where a few individuals dispersed from Nepal Himalaya into IHR and later expanded to the current population size. However, our divergence dating analysis suggest that this demographic expansion event occurred before LGM.

Palaeodistribution modelling results suggested that during LGM, the Himalayan langur distribution was spread to lower elevation central Nepal and adjoining parts of India, although the probability of distribution was less. Precipitation of the driest quarter, annual mean temperature, and precipitation seasonality were the main contributing factors in defining the suitable habitat for the Himalayan langurs. Given the cold and dry conditions during LGM at high altitudes in the Himalayas and in contrast, warmer conditions with high precipitation [74, 75] at the lower elevations (a combined effect of south west monsoon and mid-latitude westerlies), suggests that the low elevation central Nepal and adjoining parts of India could have potentially acted as refugia for these langurs. After LGM ended, the Himalayan langur distribution moved northwards; it also facilitated the movement of these langurs from central Nepal into western Himalaya where a high probability for the distribution of these langurs can be seen in the current SDM (Fig. 7). However, in our SDM for the LGM layers, we can see that there were small pockets of high probability of distribution in the western region. The asynchronous glacial advances during the LGM in the Himalayas [75, 76], could explain these pockets of high probability of distribution in the western region, however, the phylogenetic and phylogeographic analysis of the Himalayan langurs do not show signatures of refugia in the western region.

## Conclusion

We used multiple complementary methods to assess the genetic diversity, population structure and demographic history of the Himalayan langurs. In the network analysis, two distinct population clusters within the Himalayan langur were retrieved—WMP and EMP corresponding to the western clade and eastern lineages, respectively as described by Arekar et al. [52]. Interestingly, Himalayan river valleys in the IHR does not appear to affect the population subdivisions and distribution of genetic variation in the WMP, except for the populations M–Kr and Kr–Kg which showed structure across the river Karnali. The rivers in the Nepal Himalaya do act as a barrier to geneflow in the EMP. Our phylogenetic and statistical phylogeography analysis suggest a recent east to west dispersal of the Himalayan langurs. Given, the WMP is younger than EMP, it is likely that not enough time has elapsed for rivers in IHR to shape their genetic structure. Furthermore, post LGM population expansion of WMP might have also confounded their population structure. In this study, we have used only one mitochondrial Cyt-*b* gene; there might be a possibility that this gene did not capture enough variation, especially within the western population given the recent colonisation. Therefore, using markers with high substitution rates such as microsatellites and SNPs, could help us better understand the role of these river valleys in population subdivision within the Himalayan langurs.

## Methods

### Sample collection

We collected 176 fecal samples from 46 different locations in the Himalayas covering the Union Territory of Jammu and Kashmir (J&K), and the states of Himachal Pradesh (HP), Uttarakhand and Sikkim (Table S1 in [52]). Multiple samples were collected from each location. We successfully amplified a 775 bp (amplicon length) of mitochondrial Cytochrome *b* (Cyt-*b*) gene using the primer pairs Cytb_278F and Cytb_1052R. For details on DNA extraction, PCR amplification and sequencing see Arekar et al. [52].

### Phylogenetic analysis and divergence dating

The sequence files obtained were viewed and edited manually in ChromasLite v2.01 (Technelysium Pty. Ltd.). Sequences generated in this study were combined with those generated by Khanal et al. [28] and aligned using MUSCLE algorithm [77] incorporated in MEGA v7 [78]. We used jModelTest 2.1.3 [79] to pick the best model of sequence evolution. Phylogenetic reconstruction was performed using Maximum Likelihood (ML) and Bayesian methods. ML analysis was performed in RAxML7.4.2 incorporated in raxmlGUI v1.3 [80]. 1000 replicates were performed to assess support for different nodes. We used MrBayes 3.2.2 [81] to perform the Bayesian analysis. Two parallel runs, with four chains each, were run for 5 million generations with sampling frequency set to every 1000 generations. Convergence between the two runs was determined based on standard deviation of split frequencies. The program Tracer v1.6 [82] was used to determine stationarity, an effective sample size (ESS) value of > 200 for each parameter was used as a cut-off for run length. The first 25% of trees were discarded as burn-in.

For divergence time estimation we used BEAST v2.6 [83]. The sequences used for this analysis and their accession numbers are shown in Additional file 4: Table S2. The input file was prepared using BEAUti v2.6 [83]. The data was partitioned into three codon-based partitions—codon position 1 with site model K80 + G, codon position 2 with site model HKY + I and codon position 3 with site model TN93 + I. The best substitution scheme and the model of sequence evolution was selected using PartitionFinder 1.1.0 [84]. Clock model was set to uncorrelated relaxed clock lognormal. For setting the clock rate parameter, we used the rate of substitution as estimated for Human mtDNA protein coding genes [85]. The first and second codon position rate was set to 8.8 × 10^−9^ substitutions per nucleotide per year and for the third codon position, rate was set to 1.9 × 10^−8^ substitutions per nucleotide per year. The analysis was run for 100 million generations with sampling frequency of 10,000.

### Phylogeographic analyses

#### Network analysis, genetic diversity and population genetic structure

To explore the role of rivers in shaping the population genetic structure of Himalayan langurs sampling locations were divided into 10 different populations, corresponding to 10 regions in the Himalayas, demarcated by different river valleys—WestSutlej (W-S): sample locations west of the river Sutlej; Sutlej_Bhagirathi (S–B): sample locations between the rivers Sutlej and Bhagirathi; Bhagirathi_Mahakali (B–M): sample locations between the rivers Bhagirathi and Mahakali; Mahakali_Karnali (M–Kr): sample locations between the rivers Mahakali and Karnali; Karnali_KaliGandaki (Kr–Kg): sample locations between the rivers Karnali and Kali Gandaki; KaliGandaki_Marsyagandi (Kg–My): sample locations between the rivers Kali Gandaki and Marsyagandi; Marsyagandi_BudhiGandaki (My–Bg): sample locations between the rivers Marsyagandi and Budhi Gandaki; BudhiGandaki_Arun (Bg–A): sample locations between the rivers Budhi Gandaki and Arun; Arun_Tamor (A–T): sample locations between the rivers Arun and Tamor; EastTamor (E-T): sample locations east of the river Tamor (Fig. 3).

Genetic diversity indices including number of polymorphic sites (s), haplotype number (H), haplotype diversity (Hd) and nucleotide diversity (π) were calculated using DnaSP v6 [86] for each of these populations. Further, we used the median-joining (MJ) network [87] incorporated in PopART http://popart.otago.ac.nz/ [88] to build a haplotype network which graphically represents the relationship of each sample from different geographical locations. Population pairwise F_ST_ was calculated using Arlequin v3.5.2.2 and the statistical significance was tested by performing 10,000 permutations.

#### Demographic history

To infer the demographic history, we used multiple, complimentary methods. We calculated three summary statistics: Tajima’s D, Fu’s F, and Ramos-Onsins and Rozas’s R_2_ statistic for all the populations to understand the evolutionary history under different demographic scenarios using DnaSp v6 [86]. We also performed mismatch distribution analysis using the population growth-decline model to estimate the trends of population growth using DnaSp v6 [86]. We used the Bayesian Skyline Plot (BSP) analysis to estimate population size changes through time using BEAST v2.6 [83]. The best substitution model was selected using the BIC criterion in Modeltest v2.1.3 [79]. The clock model was set to strict clock and the mutation rate was set to 0.0178 mutations per site per million years [70]. The tree prior was set to BirthDeath Skyline Contemporary; and the Origin_BDSKY_Contemp.t prior was set as lognormal with mean of 2.7 and SD of 0.5. This prior sets the date of the root node, in this case the divergence between *S*. *entellus* and *S*. *schistaceus*. The analysis was run for 750 million generations with sampling every 1000 generations. We used Tracer v1.6 to check for stationarity by ensuring that the effective sample size (ESS) for each parameter was > 200. The BDSKY plot was visualised using a R script [89].

#### Statistical phylogeography

To understand the westward expansion of these langurs, we carried out a model based hypothesis testing in a Bayesian framework using Approximate Bayesian computation (ABC) analysis implemented in DIY ABC v2.1.0 [61]. ABC approximates posterior probabilities which it then uses to rank the different scenarios being considered. It first creates a prior distribution of parameter values by simulating large number of datasets under each scenario and then it uses a logistic regression method to estimate the posterior probability by picking scenarios, which are from among the simulated datasets, that are closest to the observed data [90]. For this analysis we tested three scenarios (Fig. 6), Scenario 1 hypothesised that WMP originates from EMP with no change in the effective population size; In Scenario 2 we hypothesise a founder event where few individuals from EMP colonised the western region, and the population eventually increased to the current size. and Scenario 3 assumed bottleneck in the ancestral population of WMP and EMP with increase in effective population size immediately after the divergence of the two populations. In the first two scenarios, EMP is considered as ancestral population because the phylogeny (Fig. 1) shows that WMP is nested within the EMP. The prior settings for the demographic model and the mutation model are shown in the table alongside Fig. 6. We estimated four one sample and four two sampled summary statistics. One million datasets were simulated for each scenario.

#### Niche modelling using past climate layers

For our second hypothesis, we implemented the Ecological niche modelling (ENM) approach. Here we used 217 occurrence records of the Himalayan langurs. Out of these, 104 records were from the field surveys conducted for this study, 58 occurrence records were obtained from previous studies [28, 91, 92] and 55 occurrence records were downloaded from GBIF (Global Biodiversity Information Facility) database (www.gbif.org). The occurrence records will be made available upon request. We used the MaxEnt algorithm [93] and 19 bioclimatic variables (www.worldclim.org) for the current (~ 1960–2000) and last glacial maximum (LGM) (~ 22,000 years before present, YBP) layers. Spatial resolution of LGM was resampled to 30 arcsec to match the current layers. These bioclimatic variables were clipped to the region from 68 °E to 97.4 °E and from 6.7 °N to 37 °N using ArcGIS 10.2.1. These clipped layers were then exported to ASCII format using QGIS 2.18.12. The 19 bioclimatic layers were tested for multicollinearity by calculating Pearson’s correlation coefficient (r). The layers with r ≤ |0.8| were selected for further analysis. Performance of Maxent depends on the choice of features and regularisation multiplier (RM) [93]. We tested 48 models, for the Himalayan langur dataset by employing different combinations of features and RM values (Additional file 3: Table S3) in MaxEnt v3.4.1 [93]. The model (a combination of features and RM) with the highest AUC value was selected as the best model.

ENM analysis was performed in Maxent v3.4.1 with the following modifications; Random test percentage was set to 30%, maximum number of background points was set to 10,000 and the replicates were set to 10 with replicated run type changed to Subsample. 5000 iterations were performed with the convergence threshold set to 1 × 10^−5^. Jackknife test was used to estimate the contribution of each environmental variable. The feature type was selected LQPTH with RM value 1. To overcome sampling bias, a bias file was created in R (v4.0.1) using the package ENMeval v0.3.0 [94]. The output format was chosen as Cloglog [95]. AUC values were examined to check for the predictive ability of the model.

## Supplementary Information

**Additional file 1: Figure S1.** Maximum likelihood(ML) phylogeny of Himalayan langur for mitochondrial cytochrome b (Cyt-b) gene.Numbers at the node indicate ML bootstrap (MLBS) values. Node support values of > 75 are shown. Node support values only for major clades are shown.

**Additional file 2: Figure S2.** Divergence time tree for the mitochondrial cytochrome *b *(Cyt-b) dataset constructed in BEASTv2.6. The bars at the nodes indicate the 95% credible interval. Details of the collapsed IHR node can be found in Additional file 4: Table S3. IHR = Indian HimalayanRegion; WMP = Western metapopulation; EMP = Eastern metapopulation.

**Additional file 3:Figure S3.** Response curves for top three variables of importance for Himalayan langur in the niche modelling analysis.

**Additional file 4: Table S1.** Samples used for molecular phylogenetic analysis. Samples that are marked with † were sequenced in this study. Samples with the same accession numbers are identical sequences. **Table S2.** Sequences used for divergence dating analysis in BEAST v2.6.6. Sequences that are marked with † were sequenced in this study. Sequences from sr. no. 1–66 constitutes the collapsed clade IHR in Additional file 2: Fig. S2. **Table S3.** Model selection for Maxent analysis—the table shows AUC values for different models. AUC values in bold shows the features and RM values selected.

## Data Availability

Sequences generated in this study have been deposited in GenBank under the Accession numbers OM830436–OM830486, the details of which can be found in Additional file 4: Table S1.

## Abbreviations

IHR: Indian Himalayan region
Cyt-*b*: Cytochrome-*b*
LGM: Last glacial maximum
IUCN: International Union for Conservation of Nature
YBP: Years before present
ABC: Approximate Bayesian computation
WMP: Western metapopulation
EMP: Eastern metapopulation
W-S: West Sutlej
S–B: Sutlej_Bhagirathi
B–M: Bhagirathi_Mahakali
M–Kr: Mahakali_Karnali
Kr–Kg: Karnali_KaliGandaki
Kg–My: KaliGandaki_Marsyagandi
My-Bg: Marsyagandi_BudhiGandaki
Bg–A: BudhiGandaki_Arun
A–T: Arun_Tamor
E-T: EastTamor
HKY: Hasegawa, Kishino and Yano
ML: Maximum likelihood
ESS: Effective sample size
SDM: Species distribution modelling
AUC: Area under curve
