# Correction: Geography vs. past climate: the drivers of population genetic structure of the Himalayan langur

**DOI:** 10.1186/s12862-022-02059-w

**Published:** 2022-08-23

**Authors:** Kunal Arekar, Neha Tiwari, Sambandam Sathyakumar, Mehreen Khaleel, Praveen Karanth

**Affiliations:** 1 Centre for Ecological Sciences, Indian Institute of Science, Bengaluru, India; 2 Wildlife Institute of India, Dehradun, India; 3 Wildlife Research and Conservation Foundation, Srinagar, Jammu and Kashmir India

## Correction: BMC Ecology and Evolution (2022) 22:100 10.1186/s12862-022-02054-1

Following the publication of the original article [1], we were notified that the order of the first and last name of the 3rd author was reversed. “Sathyakumar Sambandam” should be “Sambandam Sathyakumar”.

The original article [1] has been corrected.
