# Supplementary figures and images for: Geography vs. past climate: the drivers of population genetic structure of the Himalayan langur
Source: BMC Ecol Evol. 2022 Aug 15;22:100. doi: 10.1186/s12862-022-02054-1 (PMC9377076; doi:10.1186/s12862-022-02054-1)

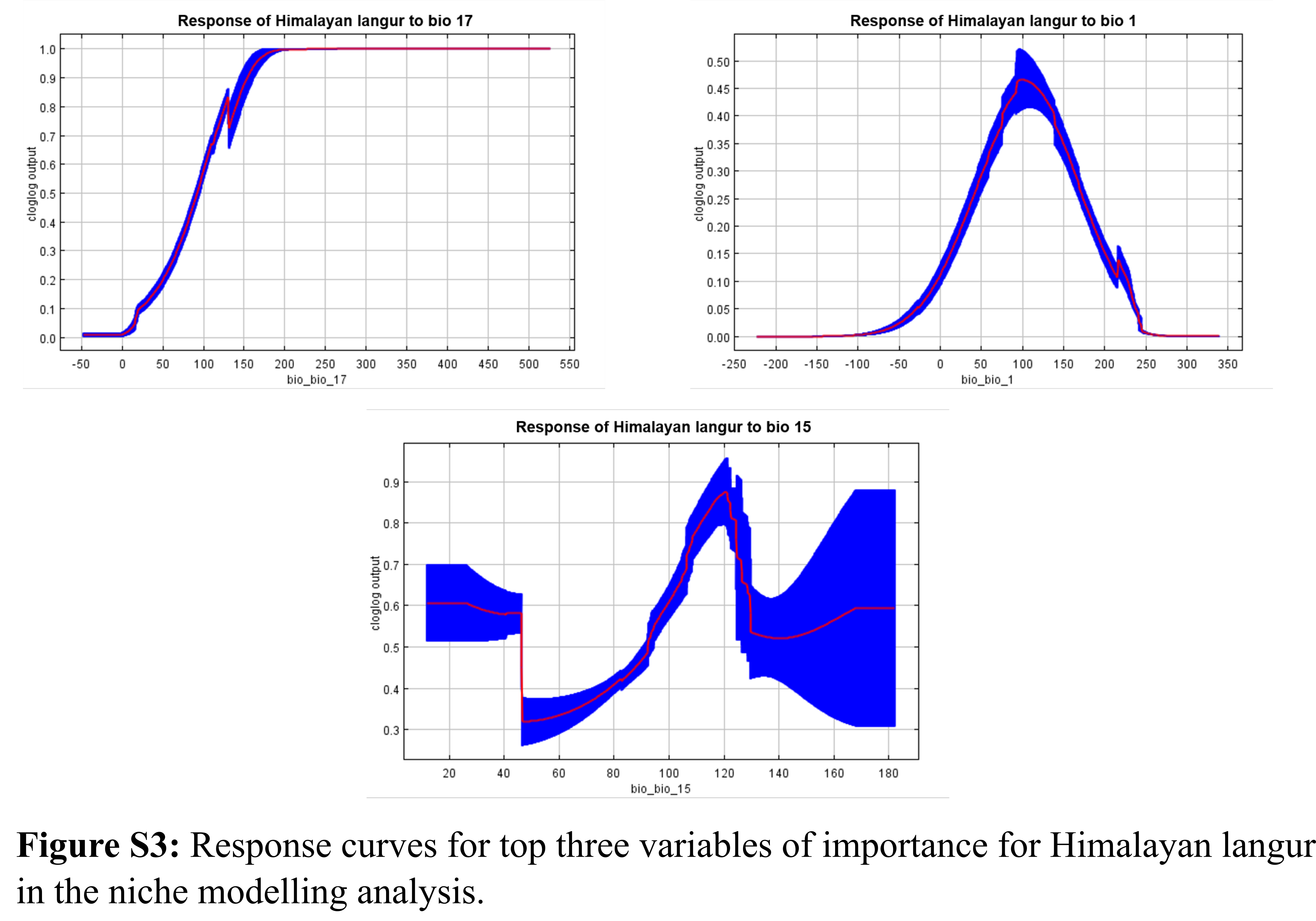

Supplement: Supplementary file 3 — Additional file 3:Figure S3. Response curves for top three variables of importance for Himalayan langur in the niche modelling analysis. [file 12862_2022_2054_MOESM3_ESM.png]
