# Supplementary material for: Geography vs. past climate: the drivers of population genetic structure of the Himalayan langur
Source: BMC Ecol Evol. 2022 Aug 15;22:100. doi: 10.1186/s12862-022-02054-1 (PMC9377076; doi:10.1186/s12862-022-02054-1)
Supplement: Supplementary file 4 — Additional file 4: Table S1. Samples used for molecular phylogenetic analysis. Samples that are marked with † were sequenced in this study. Samples with the same accession numbers are identical sequences. Table S2. Sequences used for divergence dating analysis in BEAST v2.6.6. Sequences that are marked with † were sequenced in this study. Sequences from sr. no. 1–66 constitutes the collapsed clade IHR in Additional file 2: Fig. S2. Table S3. Model selection for Maxent analysis—the table shows AUC values for different models. AUC values in bold shows the features and RM values selected. [file 12862_2022_2054_MOESM4_ESM.docx]

**Table S1:** Samples used for molecular phylogenetic analysis. Samples that are marked with † were sequenced in this study. Samples with the same accession numbers are identical sequences.

| **Samples** | **Latitude** | **Longitude** | **Accession Numbers** |
| --- | --- | --- | --- |
| 12317 | 32.52777 | 76.0836 | MT919053 |
| 13321^†^ | 30.10681 | 78.2954 | OM830436 |
| 13323^†^ | 30.10681 | 78.2954 | OM830437 |
| 13324 | 30.10681 | 78.2954 | MT919054 |
| 13325 | 31.10409 | 77.1571 | MT919046 |
| 13326^†^ | 31.10409 | 77.1571 | OM830438 |
| 13327^†^ | 31.10409 | 77.1571 | OM830439 |
| 13328^†^ | 31.10409 | 77.1571 | OM830440 |
| 13329^†^ | 31.10409 | 77.1571 | OM830441 |
| 13335 | 32.19456 | 76.1321 | MT919055 |
| 13340^†^ | 32.53511 | 76.0463 | OM830442 |
| 13345 | 32.53511 | 76.0463 | MT919048 |
| 15301^†^ | 31.1035 | 77.1542 | OM830443 |
| 15302^†^ | 31.1035 | 77.1542 | OM830444 |
| 15304^†^ | 31.1031 | 77.1526 | OM830445 |
| 15305^†^ | 31.1031 | 77.1526 | OM830446 |
| 15308 | 31.10026 | 77.2357 | MT919045 |
| 15311 | 31.34576 | 76.7839 | MT919049 |
| 15313 | 31.50311 | 76.9028 | MT919050 |
| 15314^†^ | 31.50311 | 76.9028 | OM830447 |
| 15316^†^ | 31.50311 | 76.9028 | OM830448 |
| 15318^†^ | 31.60419 | 76.4305 | OM830449 |
| 15320 | 31.60419 | 76.4305 | MT919051 |
| 15321^†^ | 31.60419 | 76.4305 | OM830450 |
| 15322^†^ | 32.53807 | 75.9544 | OM830451 |
| 15324 | 32.53407 | 75.9731 | MT919047 |
| 15325^†^ | 32.53407 | 75.9731 | OM830452 |
| 15327 | 32.5318 | 76.0176 | MT919052 |
| 17304^†^ | 30.69999 | 77.8717 | OM830453 |
| 17305 | 30.69999 | 77.8717 | MT919065 |
| 17306^†^ | 30.69999 | 77.8717 | OM830454 |
| 17310^†^ | 30.90474 | 78.3651 | OM830455 |
| 17312 | 30.90474 | 78.3651 | MT919066 |
| 17313^†^ | 30.77358 | 78.2573 | OM830456 |
| 17314^†^ | 30.77358 | 78.2573 | OM830457 |
| 17316 | 30.77358 | 78.2573 | MT919067 |
| 17317 | 30.76847 | 78.5986 | MT919068 |
| 17322^†^ | 30.76847 | 78.5986 | OM830458 |
| 17323^†^ | 30.38834 | 78.839 | OM830459 |
| 17324^†^ | 30.38834 | 78.839 | OM830460 |
| 17325^†^ | 30.38834 | 78.839 | OM830461 |
| 17326^†^ | 30.38834 | 78.839 | OM830462 |
| 17340 | 30.05083 | 79.5099 | MT919058 |
| 17342^†^ | 30.05083 | 79.5099 | OM830463 |
| 17343^†^ | 30.05083 | 79.5099 | OM830464 |
| 17344^†^ | 30.0316 | 80.1665 | OM830465 |
| 17346 | 30.0316 | 80.1665 | MT919059 |
| 17347^†^ | 30.04284 | 80.199 | OM830466 |
| 17351 | 30.04284 | 80.199 | MT919060 |
| 17352^†^ | 29.66763 | 80.2303 | OM830467 |
| 17353^†^ | 29.66763 | 80.2303 | OM830468 |
| 17355^†^ | 29.66763 | 80.2303 | OM830469 |
| 17356^†^ | 29.66763 | 80.2303 | OM830470 |
| 17357^†^ | 29.66763 | 80.2303 | OM830471 |
| 17358 | 29.66763 | 80.2303 | MT919061 |
| 17359^†^ | 29.68749 | 79.7364 | OM830472 |
| 17360^†^ | 29.68749 | 79.7364 | OM830473 |
| 17361^†^ | 29.68749 | 79.7364 | OM830474 |
| 17362 | 29.68749 | 79.7364 | MT919062 |
| 17365^†^ | 29.31781 | 79.3472 | OM830475 |
| 17366 | 29.34665 | 79.3856 | MT919064 |
| 17367^†^ | 29.34665 | 79.3856 | OM830476 |
| 17370^†^ | 29.34665 | 79.3856 | OM830477 |
| 17377^†^ | 30.05882 | 78.5111 | OM830478 |
| 17381^†^ | 30.33809 | 78.1288 | OM830479 |
| 17382^†^ | 30.33809 | 78.1288 | OM830480 |
| 17383^†^ | 30.33809 | 78.1288 | OM830481 |
| 17384 | 30.33809 | 78.1288 | MT919063 |
| 18301^†^ | 33.68274 | 74.4442 | OM830482 |
| 18302 | 33.68274 | 74.4442 | MT919056 |
| 18303^†^ | 33.95963 | 75.2961 | OM830483 |
| 18304 | 33.95963 | 75.2961 | MT919057 |
| 18327^†^ | 27.75934 | 88.5385 | OM830484 |
| 18328^†^ | 27.75934 | 88.5385 | OM830485 |
| 18329 | 27.75934 | 88.5385 | MT919069 |
| 18332^†^ | 34.1211 | 74.9944 | OM830486 |
| 271115 | 27.4594 | 87.3161 | MH271115 |
| 271115_4 | NA | NA | MH271115 |
| 271115_5 | NA | NA | MH271115 |
| 271115_6 | NA | NA | MH271115 |
| 271115_7 | NA | NA | MH271115 |
| 271116 | 27.4594 | 87.3161 | MH271116 |
| 271117 | 26.93527 | 87.3322 | MH271117 |
| 271118 | 28.1725 | 85.3503 | MH271118 |
| 271118_3 | NA | NA | MH271118 |
| 271118_4 | NA | NA | MH271118 |
| 271118_5 | NA | NA | MH271118 |
| 271118_6 | NA | NA | MH271118 |
| 271118_7 | NA | NA | MH271118 |
| 271118_8 | NA | NA | MH271118 |
| 271118_9 | NA | NA | MH271118 |
| 271118_10 | NA | NA | MH271118 |
| 271118_11 | NA | NA | MH271118 |
| 271118_12 | NA | NA | MH271118 |
| 271118_13 | NA | NA | MH271118 |
| 271119 | 28.1597 | 85.348 | MH271119 |
| 271120 | 27.8508 | 84.9913 | MH271120 |
| 271120_2 | NA | NA | MH271120 |
| 271120_3 | NA | NA | MH271120 |
| 271120_4 | NA | NA | MH271120 |
| 271121 | 27.8411 | 84.7663 | MH271121 |
| 271121_2 | NA | NA | MH271121 |
| 271122 | 28.4513 | 84.3758 | MH271122 |
| 271122_2 | NA | NA | MH271122 |
| 271123 | 28.4513 | 84.3758 | MH271123 |
| 271124 | 28.22694 | 83.6739 | MH271124 |
| 271124_3 | NA | NA | MH271124 |
| 271124_4 | NA | NA | MH271124 |
| 271124_5 | NA | NA | MH271124 |
| 271124_6 | NA | NA | MH271124 |
| 271124_7 | NA | NA | MH271124 |
| 271124_8 | NA | NA | MH271124 |
| 271124_9 | NA | NA | MH271124 |
| 271124_10 | NA | NA | MH271124 |
| 271124_11 | NA | NA | MH271124 |
| 271124_12 | NA | NA | MH271124 |
| 271124_13 | NA | NA | MH271124 |
| 271125 | 28.19166 | 83.6519 | MH271125 |
| 271126 | 28.07333 | 83.2617 | MH271126 |
| 271126_2 | NA | NA | MH271126 |
| 271127 | 28.5858 | 81.285 | MH271127 |
| 271127_2 | NA | NA | MH271127 |
| 271127_3 | NA | NA | MH271127 |
| 271128 | 28.8358 | 80.1505 | MH271128 |
| 271128_2 | NA | NA | MH271128 |
| 271128_3 | NA | NA | MH271128 |
| 271128_4 | NA | NA | MH271128 |
| 271128_5 | NA | NA | MH271128 |
| 271129 | 29.9447 | 80.9408 | MH271129 |
| 271129_2 | NA | NA | MH271129 |
| 271129_3 | NA | NA | MH271129 |
| 271129_4 | NA | NA | MH271129 |
| 271129_5 | NA | NA | MH271129 |
| 271129_6 | NA | NA | MH271129 |
| 271129_7 | NA | NA | MH271129 |
| 271129_8 | NA | NA | MH271129 |
| 271129_9 | NA | NA | MH271129 |
| 271129_10 | NA | NA | MH271129 |
| 271129_11 | NA | NA | MH271129 |
| 271129_12 | NA | NA | MH271129 |
| 271129_13 | NA | NA | MH271129 |
| 271129_14 | NA | NA | MH271129 |
| 271129_15 | NA | NA | MH271129 |
| AF959 | 26.9625 | 85.826 | AF293959 |
| *Semnopithecus entellus* | 22.8822 | 88.3997 | MT919070 |

**Table S2:** Sequences used for divergence dating analysis in BEAST v2.6.6. Sequences that are marked with † were sequenced in this study. Sequences from sr. no. 1 – 66 constitutes the collapsed clade IHR in Additional file, figure S2.

| **Sr. No.** | **Sequence** | **Accession No.** |
| --- | --- | --- |
| 1 | 12317 | MT919053 |
| 2 | 13321^†^ | OM830436 |
| 3 | 13323^†^ | OM830437 |
| 4 | 13324 | MT919054 |
| 5 | 13325 | MT919046 |
| 6 | 13326^†^ | OM830438 |
| 7 | 13327^†^ | OM830439 |
| 8 | 13328^†^ | OM830440 |
| 9 | 13329^†^ | OM830441 |
| 10 | 13335 | MT919055 |
| 11 | 13340^†^ | OM830442 |
| 12 | 13345 | MT919048 |
| 13 | 15301^†^ | OM830443 |
| 14 | 15302^†^ | OM830444 |
| 15 | 15304^†^ | OM830445 |
| 16 | 15305^†^ | OM830446 |
| 17 | 15308 | MT919045 |
| 18 | 15311 | MT919049 |
| 19 | 15313 | MT919050 |
| 20 | 15314^†^ | OM830447 |
| 21 | 15316^†^ | OM830448 |
| 22 | 15318^†^ | OM830449 |
| 23 | 15320 | MT919051 |
| 24 | 15321^†^ | OM830450 |
| 25 | 15322^†^ | OM830451 |
| 26 | 15324 | MT919047 |
| 27 | 15325† | OM830452 |
| 28 | 15327 | MT919052 |
| 29 | 17304^†^ | OM830453 |
| 30 | 17305 | MT919065 |
| 31 | 17306^†^ | OM830454 |
| 32 | 17310^†^ | OM830455 |
| 33 | 17312 | MT919066 |
| 34 | 17313^†^ | OM830456 |
| 35 | 17314^†^ | OM830457 |
| 36 | 17316 | MT919067 |
| 37 | 17317 | MT919068 |
| 38 | 17340 | MT919058 |
| 39 | 17342^†^ | OM830463 |
| 40 | 17343^†^ | OM830464 |
| 41 | 17344^†^ | OM830465 |
| 42 | 17346 | MT919059 |
| 43 | 17347^†^ | OM830466 |
| 44 | 17351 | MT919060 |
| 45 | 17352^†^ | OM830467 |
| 46 | 17353^†^ | OM830468 |
| 47 | 17355^†^ | OM830469 |
| 48 | 17356^†^ | OM830470 |
| 49 | 17357^†^ | OM830471 |
| 50 | 17358 | MT919061 |
| 51 | 17359^†^ | OM830472 |
| 52 | 17360^†^ | OM830473 |
| 53 | 17361^†^ | OM830474 |
| 54 | 17362 | MT919062 |
| 55 | 17365^†^ | OM830475 |
| 56 | 17366 | MT919064 |
| 57 | 17367^†^ | OM830476 |
| 58 | 17370^†^ | OM830477 |
| 59 | 17381^†^ | OM830479 |
| 60 | 17382^†^ | OM830480 |
| 61 | 17383^†^ | OM830481 |
| 62 | 17384 | MT919063 |
| 63 | 18301^†^ | OM830482 |
| 64 | 18302 | MT919056 |
| 65 | 18303^†^ | OM830483 |
| 66 | 18304 | MT919057 |
| 67 | 18327^†^ | OM830484 |
| 68 | 18328^†^ | OM830485 |
| 69 | 18329 | MT919069 |
| 70 | AF959 | AF293959 |
| 71 | 271115 | MH271115 |
| 72 | 271116 | MH271116 |
| 73 | 271117 | MH271117 |
| 74 | 271118 | MH271118 |
| 75 | 271119 | MH271119 |
| 76 | 271120 | MH271120 |
| 77 | 271121 | MH271121 |
| 78 | 271122 | MH271122 |
| 79 | 271123 | MH271123 |
| 80 | 271124 | MH271124 |
| 81 | 271125 | MH271125 |
| 82 | 271126 | MH271126 |
| 83 | 271127 | MH271127 |
| 84 | 271128 | MH271128 |
| 85 | 271129 | MH271129 |
| 86 | *Semnopithecus entellus* | AF293957 |
| 87 |  | JQ734727 |
| 88 |  | JQ734724 |
| 89 |  | JQ734726 |
| 90 |  | JQ734690 |
| 91 |  | JQ734725 |
| 92 |  | JQ734691 |
| 93 |  | JQ734702 |
| 94 |  | JQ734701 |
| 95 |  | JQ734733 |
| 96 |  | AF293958 |
| 97 |  | MT919070 |
| 98 |  | ON068535^†^ |
| 99 |  | ON068536^†^ |
| 100 |  | ON068538^†^ |
| 101 |  | ON068537^†^ |
| 112 | *Semnopithecus hypoleucos* | MT758700 |

**Table S3:** Model selection for Maxent analysis – The table shows AUC values for different models. AUC values in bold shows the features and RM values selected.

| **Features** | **RM value** | | | | |
| --- | --- | --- | --- | --- | --- |
|  | **1** | **1.5** | **2** | **2.5** | **3** |
| **Auto** | 0.967 | 0.961 | 0.774 | 0.868 | 0.865 |
| **L** | 0.916 | 0.919 | 0.915 | 0.910 | 0.916 |
| **LQ** | 0.907 | 0.955 | 0.957 | 0.952 | 0.962 |
| **LQP** | 0.962 | 0.959 | 0.962 | 0.959 | 0.961 |
| **LQPT** | 0.962 | 0.959 | 0.957 | 0961 | 0.955 |
| **LQPTH** | **0.968** | 0.962 | 0.961 | 0.966 | 0.960 |
| **Q** | 0.921 | 0.908 | 0.902 | 0.917 | 0.913 |
| **T** | 0.959 | 0.950 | 0.952 | 0.951 | 0.949 |
