# Supplementary material for: Geography vs. past climate: the drivers of population genetic structure of the Himalayan langur
Source: BMC Ecol Evol. 2022 Aug 15;22:100. doi: 10.1186/s12862-022-02054-1 (PMC9377076; doi:10.1186/s12862-022-02054-1)
Supplement: Supplementary file 1 — Additional file 1: Figure S1. Maximum likelihood(ML) phylogeny of Himalayan langur for mitochondrial cytochrome b (Cyt-b) gene.Numbers at the node indicate ML bootstrap (MLBS) values. Node support values of > 75 are shown. Node support values only for major clades are shown. [file 12862_2022_2054_MOESM1_ESM.pdf]

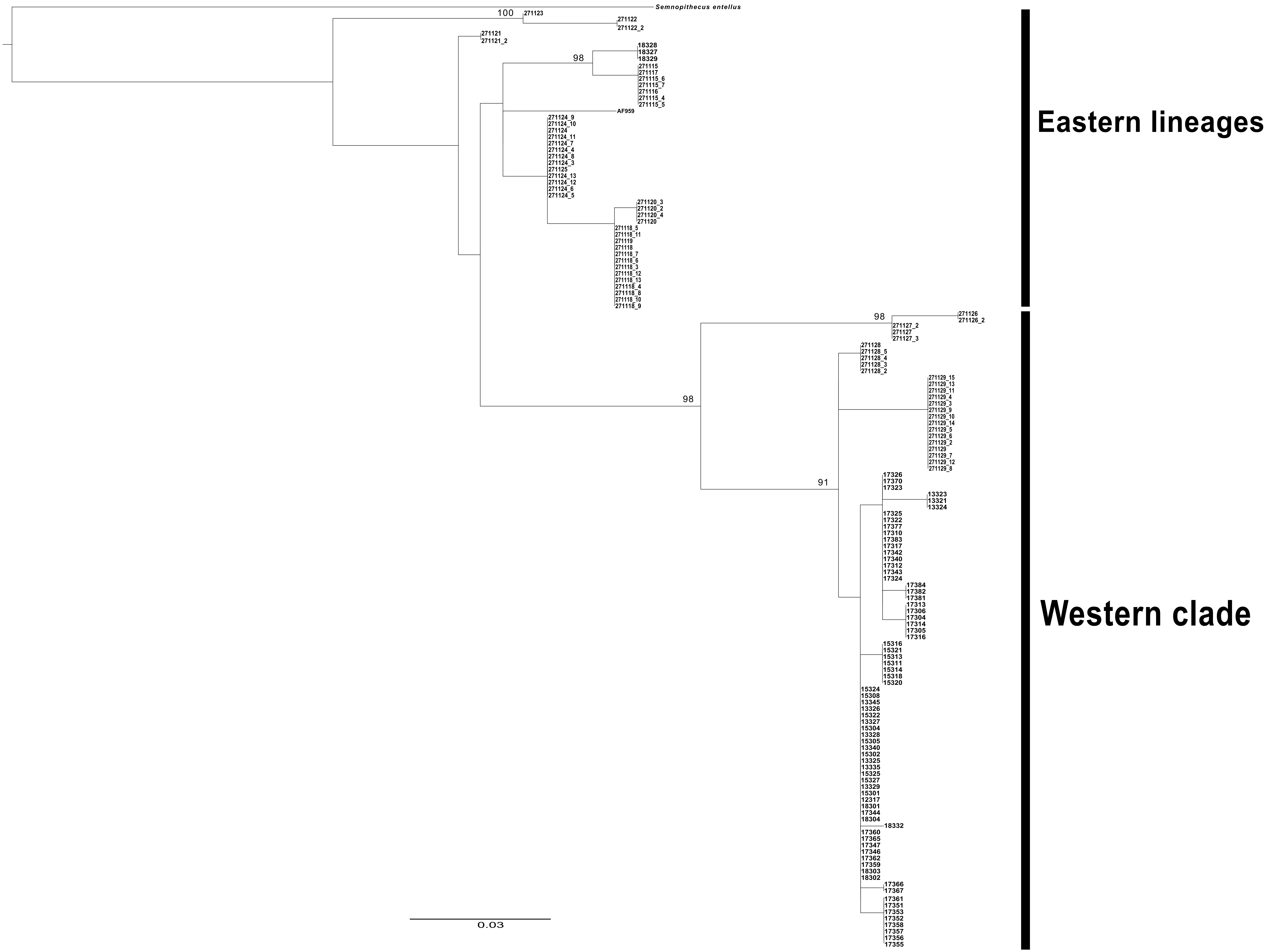

Figure S1: Maximum likelihood (ML) phylogeny of Himalayan langur for mitochondrial cytochrome b (Cyt-b) gene. Numbers at the node indicate ML bootstrap (MLBS) values.Node support values of >75 are shown. Node support values only for major clades are shown.
