# Supplementary material for: Geography vs. past climate: the drivers of population genetic structure of the Himalayan langur
Source: BMC Ecol Evol. 2022 Aug 15;22:100. doi: 10.1186/s12862-022-02054-1 (PMC9377076; doi:10.1186/s12862-022-02054-1)
Supplement: Supplementary file 2 — Additional file 2: Figure S2. Divergence time tree for the mitochondrial cytochrome b (Cyt-b) dataset constructed in BEASTv2.6. The bars at the nodes indicate the 95% credible interval. Details of the collapsed IHR node can be found in Additional file 4: Table S3. IHR = Indian HimalayanRegion; WMP = Western metapopulation; EMP = Eastern metapopulation. [file 12862_2022_2054_MOESM2_ESM.pdf]

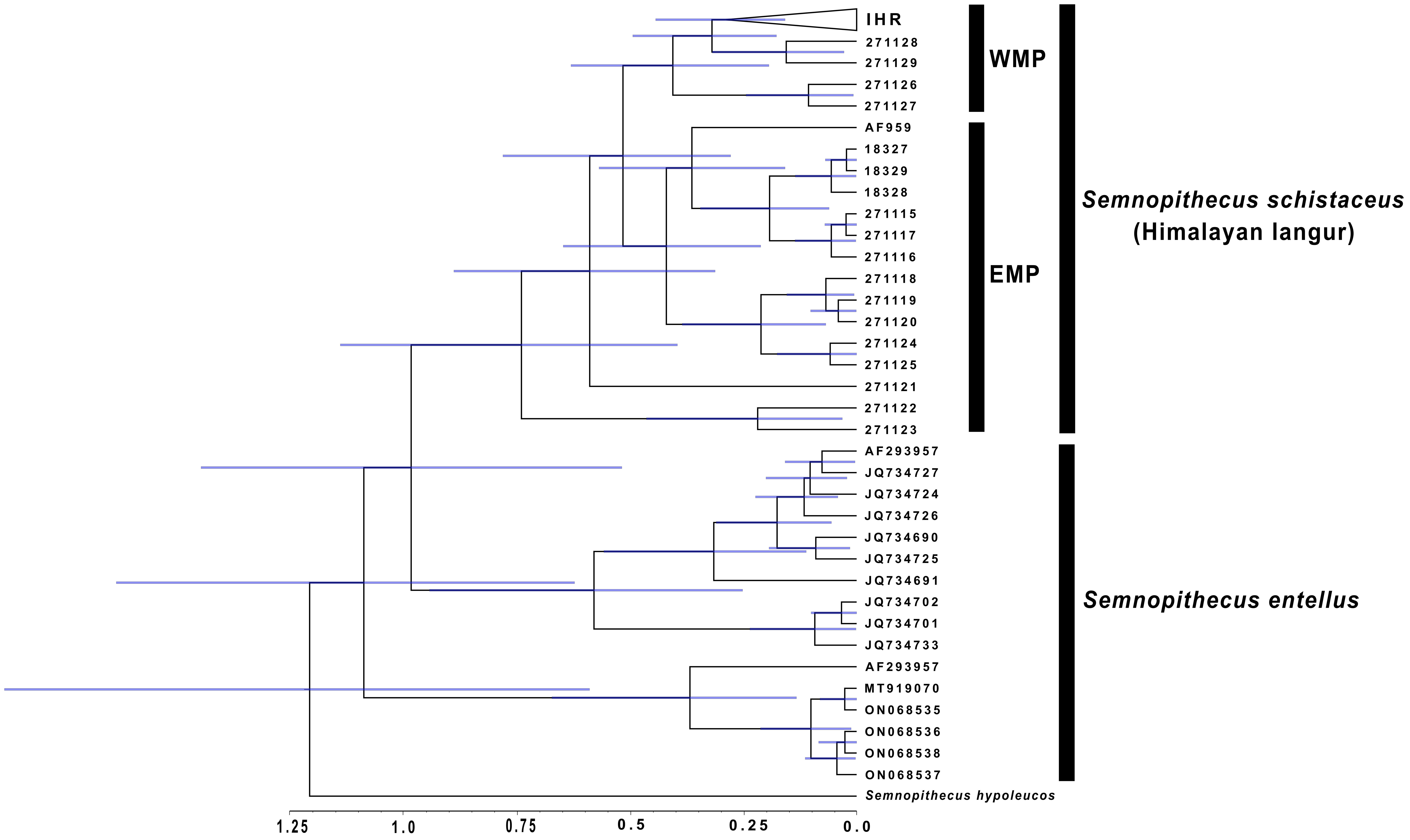

Figure S2: Divergence time tree for the mitochondrial cytochrome *b* (Cyt-b) dataset constructed in BEAST v2.6.
The bars at the nodes indicate the 95% credible interval.Details of the collapsed IHR node can be found in Additonal file 4,Table S3.
IHR = Indian Himalayan Region; WMP = Western metapopulation; EMP = Eastern metapopulation
